# Design of a ^15^N Molecular Unit to Achieve Long Retention of Hyperpolarized Spin State

**DOI:** 10.1038/srep40104

**Published:** 2017-01-09

**Authors:** Hiroshi Nonaka, Masashi Hirano, Yuki Imakura, Yoichi Takakusagi, Kazuhiro Ichikawa, Shinsuke Sando

**Affiliations:** 1 Department of Chemistry and Biotechnology, Graduate School of Engineering, The University of Tokyo, 7-3-1 Hongo, Bunkyo-ku, Tokyo 113-8656, Japan; 2 Department of Applied Chemistry, Graduate School of Engineering, Kyushu University, 744 Moto-oka, Nishi-ku, Fukuoka 819-0395, Japan; 3 Incubation Center for Advanced Medical Science, Kyushu University, 3-1-1 Maidashi, Higashi-ku, Fukuoka 812-8582, Japan; 4 Innovation Center for Medical Redox Navigation, Kyushu University, 3-1-1 Maidashi, Higashi-ku, Fukuoka 812-8582, Japan

## Abstract

Nuclear hyperpolarization is a phenomenon that can be used to improve the sensitivity of magnetic resonance molecular sensors. However, such sensors typically suffer from short hyperpolarization lifetime. Herein we report that [^15^N, D_14_]trimethylphenylammonium (TMPA) has a remarkably long spin–lattice relaxation time (1128 s, 14.1 T, 30 °C, D_2_O) on its ^15^N nuclei and achieves a long retention of the hyperpolarized state. [^15^N, D_14_]TMPA-based hyperpolarized sensor for carboxylesterase allowed the highly sensitive analysis of enzymatic reaction by ^15^N NMR for over 40 min in phophate-buffered saline (H_2_O, pH 7.4, 37 °C).

Hyperpolarization is a promising method for improving the sensitivity of magnetic resonance (MR) molecular sensors. During the polarization process, the nuclear spin state of the MR molecular sensor is polarized and the NMR sensitivity thereof is enhanced by a factor of 10^3^–10^5^ compared with that under thermally equilibrated conditions^1^.

The high sensitivity that can be achieved with hyperpolarized molecular sensors has led to the application of such systems for *in vivo* enzymatic analysis^2^,^3^,^4^,^5^. A representative example is hyperpolarized [1-^13^C]pyruvate, the use of which is being explored as a diagnostic tool for noninvasive analysis of tumor status^3^. In recent years, in addition to such biomedical applications, hyperpolarized molecules have been utilized in several research fields, including analysis of host–guest interactions^6^,^7^, monitoring of polymerization reactions^8^, and solid-surface characterization^9^. These examples show the increasing potential utility of NMR hyperpolarized techniques.

However, the hyperpolarized technique has a nonnegligible shortcoming; namely, a short lifetime of hyperpolarized state. The enhanced NMR signal decays rapidly under physiological conditions. This short lifetime has restricted the range of analysis subjects and has hampered the widespread application of this technique^2^.

To address this crucial issue, attention has focused on the development of molecular units that can retain the hyperpolarized state for longer periods^10^,^11^,^12^,^13^,^14^,^15^,^16^,^17^,^18^. The hyperpolarization lifetime is directly correlated with the spin–lattice relaxation time (*T*_1_) of nuclei. Under physiological conditions, *T*_1_ value of ^13^C nuclei in typical organic compounds used in hyperpolarized study is less than several tens of seconds; the *T*_1_ of widely used [1-^13^C]pyruvate is longer but is still in the range of 40–60 s.

Previously we developed molecular units [^15^N]trimethylphenylammonium ([^15^N]TMPA) (**1**) and [^15^N, D_9_]TMPA (**2**) (Fig. 1). The quaternary ^15^N atoms of **1** and **2** have a long *T*_1_ of 275 and 816 s (14.1 T, 30 °C, D_2_O), respectively^18^. By utilizing the unit as a platform, three types of hyperpolarized molecular sensors were designed.

Herein, we addressed the challenge of extending the hyperpolarization lifetime further. Based on the relaxation mechanism, we report an improved molecular unit, the *T*_1_ of which is 1128 ± 112 s (14.1 T, 30 °C, D_2_O). The long *T*_1_ allows that the hyperpolarized NMR signal can be detected for over 1 h.

## Results and Discussion

To achieve a long *T*_1_ for the extended hyperpolarization lifetime, we considered a relaxation mechanism (equation 1)^15^,^19^,^20^,^21^,^22^. Generally, *T*_1_ is affected by dipole–dipole (DD) relaxation, spin–rotation (SR) relaxation, chemical shift anisotropy (SA) relaxation, scalar coupling (SC) relaxation, and the other relaxation terms including dipolar relaxation from dissolved oxygen. In small to medium size molecules, the DD interaction with neighboring ^1^H atoms is one of the main factors that shorten the *T*_1_ value.


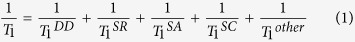


The ^15^N atom in [^15^N, D_9_]TMPA **2** has no ^1^H within two bonds, which could explain the long *T*_1_ achieved^18^. However, unexpectedly, it was found that TMPA **2** was still affected by ^15^N–^1^H DD relaxation. The role of ^15^N–^1^H DD relaxation in total relaxation, termed ^15^N–^1^H DD relaxation%, can be discussed by measurement of nuclear Overhauser enhancement (NOE, 1 + η) as previously reported (equation 2)^19^,^20^,^21^,^22^. Such ^15^N–^1^H DD relaxation% indicates whether it is possible to elongate *T*_1_ value by reducing the DD relaxation. A ^15^N–^1^H DD relaxation% value of 89.6% was determined for [^15^N]TMPA **1** (Table 1), whereas for [^15^N, D_9_]TMPA **2**, wherein all methyl ^1^H atoms were replaced with ^2^H atoms, ^15^N–^1^H DD relaxation was reduced but remained nonnegligible (21.0%). From these results, we expected that replacement of the aromatic ^1^H atoms with ^2^H, despite them being three or more bonds away from the ^15^N atom, could further extend the ^15^N *T*_1_ of the TMPA structure.


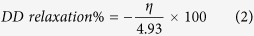


**Figure 1:**
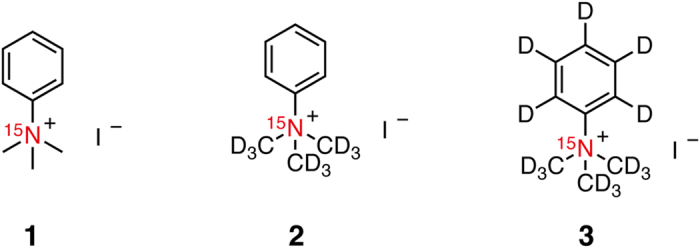
Structures of TMPA 1, 2, and 3.

**Figure 2:**
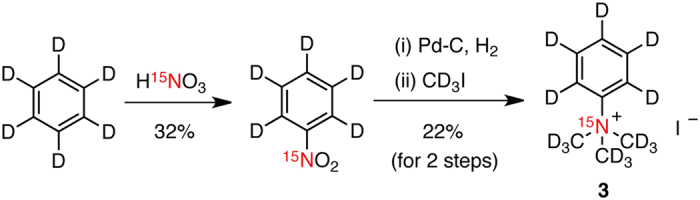
Synthesis of [^15^N, D_14_]TMPA 3.

With the above considerations in mind, we then designed fully deuterated TMPA **3** ([^15^N, D_14_]TMPA, Fig. 1). TMPA **3** was synthesized from [D_6_]benzene in three steps: nitration with [^15^N]HNO_3_, reduction through Pd/C hydrogenation, and nucleophilic displacement with CD_3_I (Fig. 2).

The *T*_1_ value of TMPA **3** was extended compared with those of both TMPA **1** and **2** (Table 1). The ^15^N *T*_1_ of TMPA **3** was determined to be 1128 ± 112 s (14.1 T, 30 °C, D_2_O) and 1177 ± 52 s (9.4 T, 30 °C, D_2_O) by using the saturation recovery method under non-degassed condition. ^15^N–^1^H DD relaxation% of TMPA **3** was quite low (1.3%) and *T*_1_ observed was longer than 1000 s in D_2_O (Table 1). This increase in the *T*_1_ of TMPA **3** could be realized by minimizing ^15^N–^1^H DD relaxation in the fully deuterated [^15^N]TMPA.

**Figure 3:**
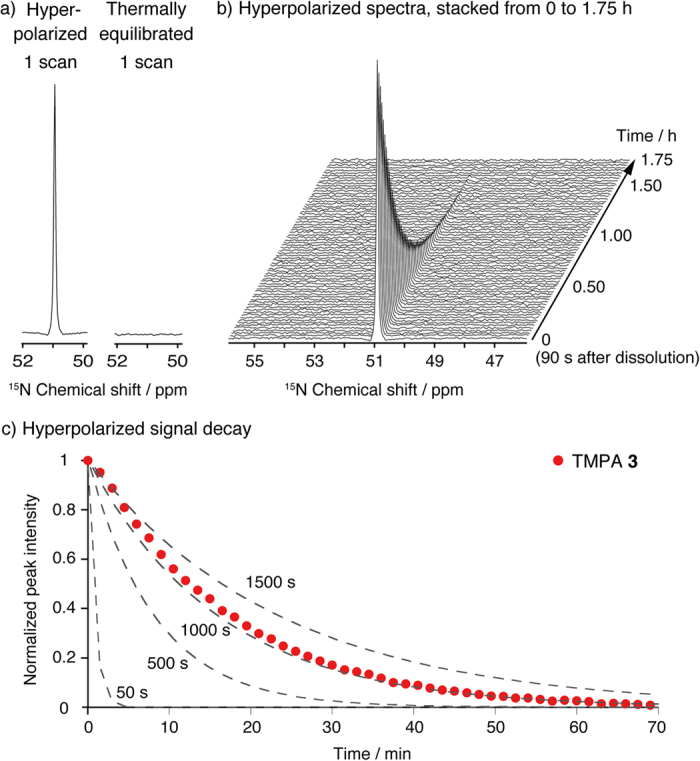
Properties of proposed platform [^15^N, D_14_]TMPA. (**a**) Single-scan ^15^N NMR spectra of hyperpolarized and thermally equilibrated [^15^N, D_14_]TMPA **3** (27 mM). (**b**) ^15^N NMR spectra of hyperpolarized **3** (27 mM) stacked from 0 (90 s after dissolution) to 1.75 h (every 90 s, pulse angle 5°). (**c**) Decay of the ^15^N NMR signal of hyperpolarized [^15^N, D_14_]TMPA **3** (red circle) and theoretical signal decay estimated by the equation^30^ on each *T*_1_ value (gray dotted line) (every 90 s, pulse angle 5°). Experiments were conducted in D_2_O containing 0.025% EDTA disodium salt.

The long *T*_1_ value for TMPA **3** led to a long retention of the hyperpolarized spin state. TMPA **3** was applied for dynamic nuclear polarization process using HyperSense (Oxford Instruments; UK) and subjected to ^15^N NMR analysis after rapid dissolution. As shown in Fig. 3a, the ^15^N NMR signal was clearly observed by measuring a single scan (5° pulse angle). The signal was enhanced by 5700 times (%P_15N_ = 1.9%, T = 298 K, B_0_ = 9.4 T) compared with that in the thermally equilibrated state. Importantly, the enhanced NMR signal of hyperpolarized **3** could be observed on a long time scale (stacked spectra in Fig. 3b), allowing detection of the hyperpolarized ^15^N signal for over 1 h under our experimental conditions (Fig. 3c). These results indicate that fully deuterated TMPA **3** has considerable potential for use as a long-lived hyperpolarization unit.

With TMPA **3** having a long ^15^N *T*_1_, we then conducted a proof-of-concept experiment by designing a long-lived hyperpolarized MR sensor.

The detection target was carboxylesterase (CE), which is a fundamental enzyme for ester hydrolysis. CE catalyzes phase I metabolism and a hydrolytic reaction by CE is used as an activation mechanism of several prodrugs^23^,^24^. Therefore, CE has been an attractive target of hyperpolarized MR molecular sensors^25^.

**Figure 4:**
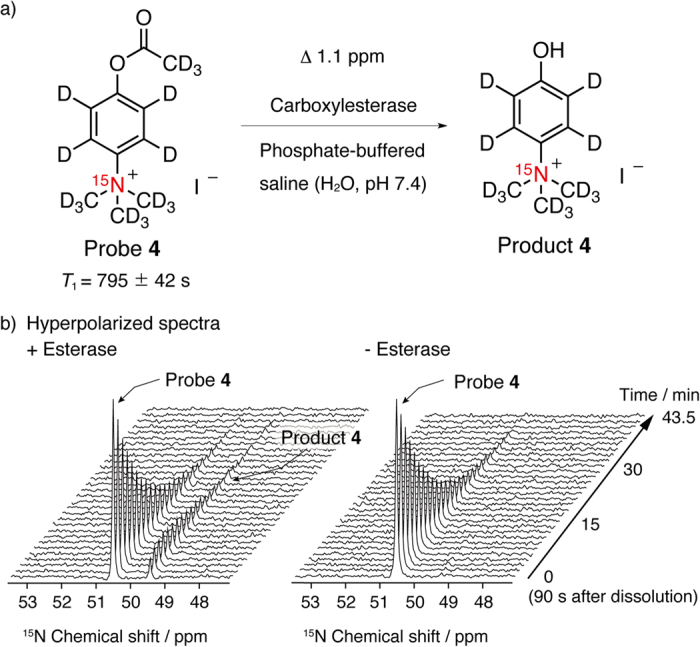
Hyperpolarized MR probe targeting carboxylesterase. (**a**) Probe **4** for sensing of carboxylesterase actvity. (**b**) Stacked single-scan ^15^N NMR spectra of hyperpolarized probe **4** (13.3 mM, every 90 s, 5° pulse angle, 0 min = 90 s after dissolution) after mixing (left) with or (right) without esterase (25 units, derived from porcine liver) in phosphate-buffered saline (H_2_O, pH 7.4).

TMPA **3** was converted into CE sensor **4**, wherein a deuterated methyl ester group was introduced at *para* position to the ^15^N nuclei (Fig. 4a). Compound **4** was synthesized from [D_6_]phenol in four steps (Supplementary Figure S1). The *T*_1_ values of sensor **4** were 795 ± 42 s (9.4 T, 30 °C, D_2_O) and 602 ± 52 s (9.4 T, 37 °C, 90% H_2_O + 10% D_2_O). These *T*_1_ values were longer than those of previously reported [^15^N, D_9_]TMPA-based esterase probe (Supplementary Figure S2; 570 ± 86 s, 9.4 T, 30 °C, D_2_O; 450 ± 15 s, 9.4 T, 37 °C, 90% H_2_O + 10% D_2_O) under the same experimental conditions^18^.

Hyperpolarized molecule **4** worked as a chemical shift-switching sensor for detection of CE activity in phosphate-buffered saline (H_2_O, pH 7.4) (Fig. 4a and b). The high sensitivity of the technique meant that ^15^N NMR spectra of hyperpolarized sensor **4** could be detected by a single-scan measurement (50.5 ppm). In the presence of CE derived from porcine liver, hyperpolarized sensor **4** afforded a new ^15^N signal 1.1 ppm upfield of the parent signal (49.4 ppm). This new peak was assigned as that of hydrolyzed product **4** by thermally equilibrated NMR analysis, suggesting that this hyperpolarized probe is suitable for use as a molecular sensor for CE activity. The 1.1 ppm change in ^15^N chemical shift may need to be enlarged especially for *in vivo* application^26^,^27^. However, it is sufficient to monitor enzymatic reaction *in vitro*. Thanks to the long *T*_1_, this enzymatic reaction could be tracked for approximately 40 min, which is longer than is possible for typical hyperpolarized molecular sensors (tens of seconds or a few minutes).

In summary, we have developed a molecular unit that is characterized by a remarkably long retention of hyperpolarized signal. The developed [^15^N, D_14_]TMPA unit showed a long *T*_1_ value (1128 s, 14.1 T, 30 °C, D_2_O) on its ^15^N nuclei and long-lived hyperpolarization. To our knowledge, this *T*_1_ is the longest among the soluble ^13^C or ^15^N small organic molecules utilized in hyperpolarization studies^10^,^11^,^18^. Such a long *T*_1_ value allows the molecule to be used in a diverse range of applications. [^15^N, D_14_]TMPA can work as a platform for designing a variety of hyperpolarized molecular sensors as shown in this study. Furthermore, it is feasible to use [^15^N, D_14_]TMPA as a hyperpolarized MRI tracer for post-polarization labelling^28^ of biomolecules such as peptides, proteins, and synthetic ligands, while conventional hyperpolarized units are not suitable for use in labelling techniques because of their limited hyperpolarization lifetimes. The [^15^N, D_14_]TMPA unit, in combination with a smart cross-polarization technique to boost the polarization level^29^, might realize such applications.

In this study, the long hyperpolarized lifetime was achieved by minimizing ^15^N-^1^H DD *T*_1_-relaxation, suggesting that it is possible to design a longer-lived hyperpolarization unit by carefully investigating *T*_1_ relaxation contributions. The knowledge and information obtained by these trials would provide important guidelines for the design of new hyperpolarized probes. Further work along these lines is under way in our laboratories.

## Methods

### General information on synthesis

Reagents and solvents were purchased from standard suppliers and used without further purification. Gel permeation chromatography (GPC) was performed on JAIGEL GS310 using a JAI Recycling Preparative HPLC LC-9201. NMR spectra were measured using a Bruker Avance III spectrometer (400 MHz for ^1^H) and a JEOL ECS400 spectrometer. Acetone-d_6_ in methanol (2.15 ppm) was used as the internal standard for ^2^H NMR. Chloroform-d_1_ (77.0 ppm) or methanol-d_4_ (49.0 ppm) was used as the internal standard for ^13^C NMR. Choline chloride-^15^N (43.4 ppm) was used as the external standard for ^15^N NMR. Mass spectra (MS) were measured using a JEOL JMS-HX110A (FAB).

### Synthesis of [^15^N, D_5_]4-nitrobenzene

[^15^N]Nitric acid 40w/w% (3.00 mL, 23.8 mmol) was added dropwise to a solution of benzene-d_6_ (2.00 g, 23.8 mmol) in sulfuric acid (3.2 mL) on ice. The mixture was stirred at 50 °C for 1 h. Ice and water were added to the mixture. After removing the aqueous phase, the resulting organic phase was purified by distillation using Kugelrohr apparatus to give [^15^N, D_5_] 4-nitrobenzene as a pale yellow liquid (980 mg, 32%): ^13^C NMR (CDCl_3_, 100 MHz) *δ* = 122.7 (^1^*J*_CD_ = 26 Hz), 128.5 (^1^*J*_CD_ = 25 Hz), 133.9 (^1^*J*_CD_ = 25 Hz), 147.7 (d, ^1^*J*_CN_ = 15 Hz); ^15^N NMR (CDCl_3_, 40 MHz) *δ* = 366.5.

### Synthesis of [^15^N, D_14_]trimethylphenylammonium (3)

[^15^N, D_5_]4-Nitrobenzene (500 mg) in methanol (5 mL) was stirred under hydrogen at r.t. for 2 h in the presence of 10 wt.% palladium on activated carbon (50 mg). The solution was filtered, and the filtrate was evaporated to give the reductant. Subsequently, [D_3_]iodomethane (938 μL, 15.1 mmol) was added to a solution of the residue (326 mg, 3.29 mmol) and *N*,*N*′-diisopropylethylamine (2.29 mL, 13.2 mmol) in dry DMF (7 mL). The mixture was stirred at room temperature for 35 h. After evaporation, EtOAc was added to the crude residue to produce a white precipitate. The resulting precipitate was filtered and purified using GPC (eluent: methanol) to give **3** as a white powder (235 mg, 22% for 2 steps): ^2^H NMR (MeOH, 61 MHz) *δ* = 3.7 (9 × ^2^H), 7.7 (2 × ^2^H + 1 × ^2^H), 8.0 (2 × ^2^H); ^13^C NMR (CD_3_OD, 100 MHz) *δ* = 55.2–56.1 (m), 119.6 (^1^*J*_CD_ = 25 Hz), 129.7–130.1 (m), 147.0 (d, ^1^*J*_CN_ = 7 Hz); ^15^N NMR (D_2_O, 40 MHz) *δ* = 50.9; HRMS(FAB): m/z calc. for C_9_D_14_^15^N [M−I]^+^ = 151.1975, found = 151.1971.

### *T*_1_ measurements

All *T*_1_ measurements were performed at thermal equilibrium. The *T*_1_ measurements of MR probes were performed using a JEOL ECA 600 (14.1 T, 30 °C, 200–500 mM) and JEOL ECS 400 (9.4 T, 30 °C, 200–400 mM) by saturation recovery method under non-degassed condition.

### NOE (1 + η) measurements

The NOE (1 + η) of MR probes **1–3** was measured on a JEOL ECS 400 (9.4 T, 30 °C). After measuring the ^15^N NMR spectra with or without NOE, the value η was determined using the following equation 3^19^,^20^:


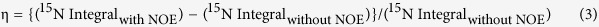


### General information on DNP–NMR measurements

Tris{8-carboxyl-2,2,6,6-tetra[2-(1-hydroxyethyl)]-benzo(1,2-d:4,5-d′)bis(1,3)dithiole-4-yl}methyl sodium salt (Ox63 radical, GE Healthcare) and the ^15^N-labelled sample were dissolved in a 1:1 solution of D_2_O (99.9%, D):dimethyl sulfoxide-d6 (99.8%, D) (final concentration of Ox63 10–15 mM). The sample was submerged in liquid helium in a DNP polarizer magnet (3.35 T) (HyperSense, Oxford Instruments). The transfer of polarization from the electron spin on the radical to the ^15^N nuclear spin on the probe was achieved using microwave irradiation at 94 GHz and 100 mW under 2.8 mbar at 1.4 K. After polarization, samples were dissolved in D_2_O containing 0.025% EDTA disodium salt or an appropriate buffer heated to ~185 °C (pressurized to 10 bar)^18^. The DNP-NMR measurement was performed using a Japan Redox JXI-400Z spectrometer (9.4 T).

### Time course analysis of hyperpolarized [^15^N, D_14_]TMPA (3)

The hyperpolarized [^15^N, D_14_]TMPA (final concentration 27 mM) was dissolved in D_2_O containing 0.025% EDTA disodium salt (4 mL). The solution was passed through two anion exchange cartridges (Grace: USA) to remove the remaining Ox63 radical, transferred to a 10 mm NMR tube, and subjected to ^15^N NMR analysis (flip angle 5°, repetition time 90 s).

### Carboxyesterase sensing by probe 4

The hyperpolarized probe **4** (final concentration 13.3 mM) was dissolved in PBS (H_2_O, pH 7.4) containing 0.025% EDTA disodium salt (4 mL). The solution was passed through two anion exchange cartridges (Grace: USA) to remove the remaining Ox63 radical. The resulting solution was mixed with carboxylesterase (Sigma-Aldrich E2884, 25 units), transferred to a 10 mm NMR tube, and subjected to ^15^N NMR analysis (flip angle 5°, repetition time 90 s).

## Additional Information

**How to cite this article**: Nonaka, H. *et al*. Design of a ^15^N Molecular Unit to Achieve Long Retention of Hyperpolarized Spin State. *Sci. Rep.* **7**, 40104; doi: 10.1038/srep40104 (2017).

**Publisher's note:** Springer Nature remains neutral with regard to jurisdictional claims in published maps and institutional affiliations.

## Supplementary Material

Supplementary Information

## Figures and Tables

**Table 1:** Relaxation properties of TMPA 1, 2, and 3.

	*T*_1_ (14.1 T)^a^	*T*_1_ (9.4 T)^a^	NOE (1 + η)^b^ for ^15^N	^15^N–^1^H DD-relaxation%^b^
1	275 ± 11 s^c^	262 ± 16 s	−3.42 ± 0.09	89.6 ± 1.9%
2	816 ± 15 s^c^	880 ± 38 s	−0.04 ± 0.02	21.0 ± 0.5%
3	1128 ± 112 s	1177 ± 52 s	0.94 ± 0.04	1.3 ± 0.8%

^a^*T*_1_ values were determined by using the saturation recovery method (14.1 T and 9.4 T, 30 °C, D_2_O).

^b^NOE (1 + η) and ^15^N–^1^H DD-relaxation% were determined on 9.4 T NMR analysis (30 °C, D_2_O).

^c^*T*_1_ values reported in ref. 18.
