# Supplementary material for: Design of a 15N Molecular Unit to Achieve Long Retention of Hyperpolarized Spin State
Source: Sci Rep. 2017 Jan 9;7:40104. doi: 10.1038/srep40104 (PMC5220364; doi:10.1038/srep40104)
Supplement: Supplementary Information [file srep40104-s1.pdf]

Supplementary Information

For

Design of a ^{15}N Molecular Unit to Achieve Long Retention of Hyperpolarized Spin State

Hiroshi Nonaka[1]*, Masashi Hirano[2], Yuki Imakura[1], Yoichi Takakusagi[3], Kazuhiro Ichikawa[3,4] and Shinsuke Sando[1]*

[1]*Department of Chemistry and Biotechnology, Graduate School of Engineering, The University of Tokyo, 7-3-1 Hongo, Bunkyo-ku, Tokyo 113-8656, Japan.*

[2]*Department of Applied Chemistry, Graduate School of Engineering, Kyushu University, 744 Moto-oka, Nishi-ku, Fukuoka 819-0395, Japan.*

[3]*Incubation Center for Advanced Medical Science, Kyushu University, 3-1-1 Maidashi, Higashi-ku, Fukuoka 812-8582, Japan.*

[4]*Innovation Center for Medical Redox Navigation, Kyushu University, 3-1-1 Maidashi, Higashi-ku, Fukuoka 812-8582, Japan.*

Supplementary Figures:

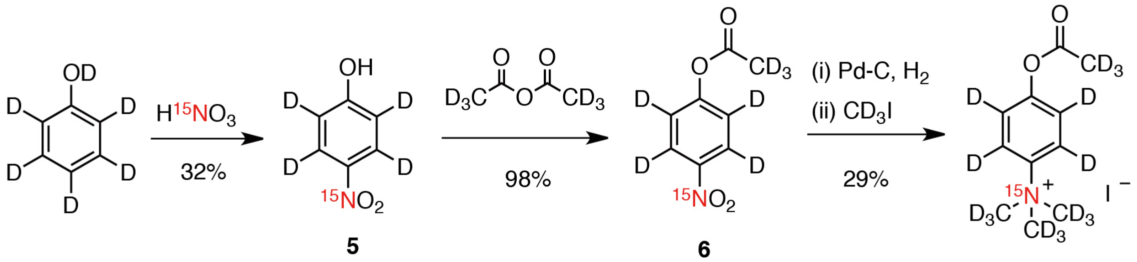

5 6 4

Supplementary Figure S1 | Synthetic scheme of esterase probe 4.

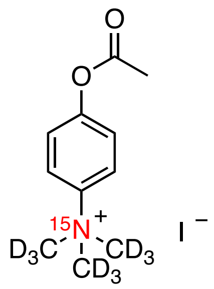

Supplementary Figure S2 | Chemical structure of our previously reported [^{15}N,D_9]TMPA-based esterase probe (ref. 18).

Synthesis

General. Reagents and solvents were purchased from standard suppliers and used without further purification. Gel permeation chromatography (GPC) was performed on JAIGEL GS310 using a JAI Recycling Preparative HPLC LC-9201. NMR spectra were measured using a Bruker Avance III spectrometer (400 MHz for ^{1}H) and a JEOL ECS400 spectrometer. Chloroform-d_1 (7.26 ppm), methanol-d_4 (3.31 ppm), or D_2O (4.79 ppm) was used as the internal standard for ^{1}H NMR. Acetone-d_6 in chloroform (2.15 ppm) or acetone-d_6 in methanol (2.15 ppm) was used as the internal standard for ^{2}H NMR. Chloroform-d_1 (77.0 ppm), methanol-d_4 (49.0 ppm), or methanol in D_2O (49.5 ppm) was used as the internal standard for ^{13}C NMR. Choline chloride-^{15}N (43.4 ppm) was used as the external standard for ^{15}N NMR. Mass spectra (MS) were measured using a JEOL JMS-HX110A (FAB) and a Thermo Scientific Exactive (ESI) .

Synthesis of 5.

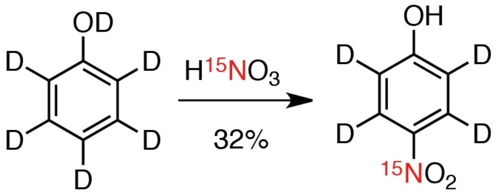

5

[^{15}N]Nitric acid 40w/w% (2.00 mL, 15.8 mmol) was added dropwise to a solution of [D_6]phenol (1.46 g, 24.7 mmol) in acetic acid (7.0 mL). The mixture was stirred at room temperature for 12 h. Water was added and the mixture was extracted with $CHCl_3$. The organic phase was washed with brine and dried over sodium sulfate. After removing the solvent *in vacuo*, the resulting residue was purified using silica gel column chromatography (eluent: hexane:EtOAc = 15:1) to give **5** as a yellow solid (669 mg, 32%): ^{13}C NMR (CD_3OD, 100 MHz) δ = 116.1 ($^1J_{CD}$ = 25 Hz), 126.6 ($^1J_{CD}$ = 25 Hz), 141.5 (d, $^1J_{CN}$ = 15 Hz), 165.0; ^{15}N NMR (CD_3OD, 40 MHz) δ = 366.9; HRMS(ESI): m/z calc. for $C_6HD_4{}^{15}NNaO_3$ $[M+Na]^+$ = 167.0383, found = 167.0394.

Synthesis of 6.

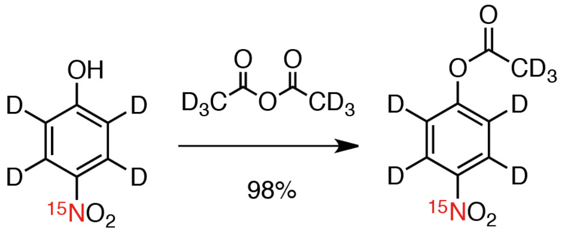

5 6

[D_6]Acetic anhydride (1.30 mL, 13.8 mmol) was added to a solution of **5** (510 mg, 3.54 mmol) in pyridine (3.5 mL). The mixture was stirred at room temperature under a nitrogen atmosphere. After 20 h, EtOAc was added and the mixture was washed with saturated aqueous $NaHCO_3$. The resulting organic phase was dried over sodium sulfate and evaporated. The resulting oil was co-evaporated with toluene to give **6** as a yellow solid (653 mg, 98%): ^{13}C NMR ($CDCl_3$, 100 MHz) δ = 20.1-20.5 (m), 121.9 ($^1J_{CD}$ = 26 Hz), 124.7 ($^1J_{CD}$ = 26 Hz), 145.1 (d, $^1J_{CN}$ = 16 Hz), 155.2, 168.2; ^{15}N NMR ($CDCl_3$, 40 MHz) δ = 363.6; HRMS(ESI): m/z calc. for $C_8D_7{}^{15}NNaO_4$ $[M+Na]^+$ = 212.0677, found = 212.0691.

Synthesis of probe 4.

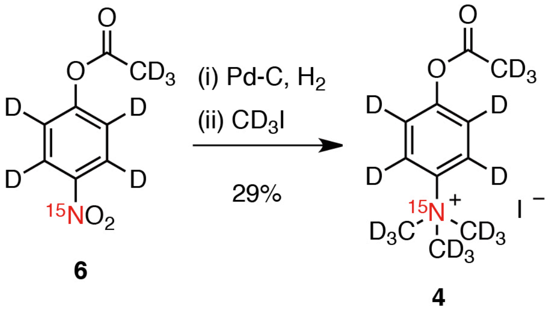

Compound **6** (594 mg, 3.14 mmol) in EtOAc (20 mL) was stirred under hydrogen at room temperature in the presence of 10 wt.% palladium on carbon (38 mg). After 24 h, this solution was filtered through celite, and the filtrate was evaporated to give the reductant. Subsequently, [D_3]iodomethane (920 µL, 14.8 mmol) was added to a solution of the residue and *N,N'*-diisopropylethylamine (850 mg, 6.59 mmol) in dry DMF (15 mL). The mixture was stirred at r.t. under a nitrogen atmosphere. After 48 h, [D_3]iodomethane (700 µL, 11.3 mmol) was added to the mixture and stirred at r.t. under a nitrogen atmosphere for 12 h. After removing the solvent *in vacuo*, the resulting oil was co-evaporated with toluene. Methanol was added to the residue to produce a white precipitate. The precipitate was filtered and washed with methanol to give **4** as a white powder (306 mg, 29% for 2 steps): ^{13}C NMR (D_2O, 100 MHz) δ = 20.3-20.6 (m), 56.5-57.0 (m), 121.9 ($^1J_{CD}$ = 25 Hz), 123.9 ($^1J_{CD}$ = 26 Hz), 144.5 (d, $^1J_{CN}$ = 8 Hz), 151.5, 173.3; ^{15}N NMR (D_2O, 40 MHz) δ = 50.5; HRMS(FAB): m/z calc. for $C_{11}D_{16}O_2{}^{15}N$ $[M{-}I]^+$ = 211.2156, found = 211.2156.
